# Exploring Biomarkers and Regulatory Mechanisms Associated with Lytic Cell Death in Allergic Rhinitis Based on Transcriptome Analysis

**DOI:** 10.3390/biomedicines14061284

**Published:** 2026-06-04

**Authors:** Rui Dong, Zhishan Dong, Zhigang Geng, Lei Lu, Yongjin Ji, Jinmei Xue

**Affiliations:** 1 Department of Otolaryngology, Head & Neck Surgery, Second Hospital of Shanxi Medical University, Taiyuan 030001, China; dongrui@sxmu.edu.cn (R.D.);; 2 Shanxi Key Laboratory of Rapid Diagnosis and Precision Treatment of Airway Allergic Diseases, Taiyuan 030001, China; 3 Shanxi Provincial Center for Disease Control and Prevention, Taiyuan 030032, China

**Keywords:** allergic rhinitis, lytic cell death, biomarkers, machine learning, immune infiltration

## Abstract

**Background:** Allergic rhinitis (AR) is a common inflammatory disorder with an unclear role of lytic cell death (LCD). This study aimed to identify LCD-associated genes associated with AR and investigate their underlying regulatory pathways. **Methods:** Transcriptomic data from AR patients (GSE19187, GSE206149) were retrieved from public repositories, and LCD-associated genes were collected from the literature. A combination of differential expression analysis, machine learning techniques, validation of expression levels, and ROC curve analysis was employed to screen for biomarkers. These biomarkers were then subjected to comprehensive functional characterization via GSEA, subcellular localization prediction, immune infiltration profiling, construction of molecular regulatory networks, and drug prediction. Finally, clinical relevance was confirmed through expression levels in patient specimens. **Results:** Two key indicators, ALOX15 and TIMP1, were successfully pinpointed. GSEA revealed significant enrichment of ALOX15 and TIMP1 in several biological processes, specifically chromatin organization, immune system response, and extracellular substance transport. Subcellular distribution studies showed that ALOX15 predominantly localized in the cytosol and plasma membrane, while TIMP1 was mainly detected extracellularly. Immune infiltration studies demonstrated notable modifications in seven immune cell populations, with significant associations with megakaryocyte–erythroid progenitors and conventional dendritic cells. Based on these findings, a regulatory network composed of transcription factors and microRNAs was established, and several potential therapeutic candidates (e.g., quercetin) were identified through prediction. Consistent with predictions, mRNA expression levels of both genes were significantly upregulated in the AR group compared to controls (*p* < 0.01), confirming reliability. **Conclusions:** In summary, ALOX15 and TIMP1 were identified as exploratory biomarkers associated with AR, providing preliminary insights into its molecular mechanisms and potential therapeutic implications.

## 1. Introduction

Characterized by nasal inflammation that persists over time, allergic rhinitis occurs when the immune system, responding to airborne allergens, releases immunoglobulin E, initiating an inflammatory process in the nasal lining. Globally, its prevalence ranges from 30% to 40% and is still increasing, imposing a heavy burden on quality of life and medical costs [1]. Characterized by type 2 inflammatory profiles, AR is a condition that stems from the intricate interaction between genetic predisposition and environmental triggers including allergen contact and atmospheric pollution [2]. In clinical practice, AR is typically diagnosed based on characteristic symptoms, allergen testing, and additional examinations; however, these methods often fail to precisely reflect the individual’s inflammatory subtype and disease progression. While conventional treatments like intranasal corticosteroids and antihistamines can alleviate symptoms, they generally focus on relieving manifestations rather than addressing the root cause, and symptoms may recur once therapy is stopped [3]. Emerging as promising alternatives for managing AR, novel biologic agents like Stapokibart have shown potential; however, the lack of diverse patient populations—with most early studies focusing on Chinese participants—has hindered a full evaluation of their long-term effectiveness [4]. Allergen-specific immunotherapy (AIT) represents the sole potential disease-modifying approach for AR; however, its clinical application is constrained by a prolonged treatment duration, elevated costs, and substantial inter-individual variability in therapeutic outcomes [5]. Consequently, the in-depth investigation of the complex molecular mechanisms underlying AR, alongside the identification of biomarkers capable of distinguishing clinical subtypes and predicting treatment responses via bioinformatics methodologies, carries significant scientific and clinical implications. These endeavors seek to elucidate pathogenic mechanisms comprehensively at the molecular level, while simultaneously promoting the establishment of innovative early diagnostic modalities and targeted therapeutic interventions.

Recent studies suggested that lytic cell death potentially exerted regulatory effects on the occurrence and progression of allergic rhinitis [6]. Defined as a group of cell death patterns, lytic cell death is mainly marked by the breakdown of cell membrane structure [7]. Previous studies have demonstrated that, under physiological conditions, lytic cell death serves as an essential mechanism through which for the body to eliminate pathogenic infections. Under pathological conditions, excessive cell lysis promotes the release of intracellular substances such as high-mobility group box 1 (HMGB1) and damage-associated molecular patterns (DAMPs) [8]. These released molecules can activate innate immune responses and the progression of inflammatory cascades, which might correlate with the pathological processes of various diseases [9,10]. In the context of AR, prolonged allergen exposure may induce lytic cell death in nasal mucosal epithelial cells and multiple other cell populations. Such lytic cell death could facilitate the release of DAMPs. For example, HMGB1 might initiate T helper 2 (Th2) immune responses through specific receptors, while interleukin-33 (IL-33) may potentially promote the activation of both group 2 innate lymphoid cells (ILC2s) and Th2 cells. The released DAMPs could act as a potential intermediate link between the initial allergic response and the progression of chronic Th2 inflammation, which may further exacerbate nasal inflammation and hyperresponsiveness [11,12,13]. A thorough investigation into the mechanisms behind lytic cell death in AR will not only aid in understanding the immunopathological foundation of the disease but also offer valuable theoretical support and potential therapeutic targets for new approaches that aim to disrupt the chronic inflammatory process of AR and break the cycle of Th2 immune dominance. Notably, the core molecular mechanisms of LCD are highly conserved across various diseases. Studies have demonstrated that, regardless of the pathogenic trigger, LCD is universally mediated by membrane pore-forming proteins such as GSDMD, MLKL, and NINJ1, which drive cell membrane rupture accompanied by the release of damage-associated molecular patterns (DAMPs), thereby initiating the innate immune response [14,15,16,17]. This conserved execution mechanism suggests that LCD-associated genes hold great potential for cross-disease research. In addition, Zhang et al. [18] constructed an LCD-associated gene set by integrating multiple cell death modalities including pyroptosis, necroptosis, and PANoptosis. The screening criteria for this gene set were based on the common morphological characteristics and core regulatory pathways shared by diverse cell death types, rather than the specific pathological processes of atherosclerosis, which provides a solid research foundation for our identification of potential LCD-associated genes in allergic rhinitis (AR).

In this study, our research team downloaded AR-related transcriptomic data from public databases and further collected LCD-associated genes from published academic literature. It should be clarified that our research strategy aims to explore potential LCD-associated molecular signatures and their regulatory associations in the pathological process of AR through intersection analysis at the transcriptomic level, rather than directly verifying the occurrence of LCD events in the nasal mucosa of AR patients. We further performed differential expression analysis to screen potential AR-related genes, and then identified exploratory biomarkers for AR via integrative bioinformatics and machine learning analyses. Moreover, we deeply explored the molecular mechanisms of these biomarkers in AR, clarified their relevant biological pathways and interactions with the immune microenvironment, and ultimately laid a foundation for the development of novel diagnostic and therapeutic strategies for AR.

## 2. Materials and Methods

### 2.1. Data Collection

AR-related datasets were retrieved from the Gene Expression Omnibus (GEO). The training set, GSE19187 (platform GPL6244, expression profiling by array), comprised nasal epithelial tissue samples from 14 AR patients and 11 healthy controls, and was utilized for differential expression analysis. Another dataset, the validation set, GSE206149 (platform GPL16791, high-throughput sequencing), was composed of nasal brush samples from 78 AR patients and 15 healthy controls, and was used to confirm the gene expression patterns identified. Moreover, a collection of 599 LCD-associated genes was constructed based on a prior research article [14] (Supplementary Table S1).

### 2.2. Identification of Candidate Genes

Raw expression data were processed with routine background correction and quantile normalization, and low-expression probes were filtered out. Probes were annotated and matched to gene symbols based on the platform annotation file. Expression signals were integrated when multiple probes mapped to the same gene, and missing values in the expression matrix were imputed using a standard conventional approach. Differential expression analysis (|log2FoldChange (FC)| > 0.5 and *p* < 0.05) was performed on the training dataset to identify candidate genes by comparing AR and control samples (AR vs. control) using the “limma” package (v 3.58.1) [19]. First, multiple testing correction was conducted using the Benjamini–Hochberg (BH) method, and differentially expressed genes (DEGs) were then identified with the thresholds of |log2FoldChange (FC)| > 0.5 and *p* < 0.05. The resulting differentially expressed genes (DEGs) were visualized via a volcano plot created using “ggplot2” (v 3.5.1) [20] and “ggrepel” (v 0.9.5), with the top 10 upregulated and downregulated DEGs annotated according to their |log2FC| values. Additionally, the expression profiles of these DEGs were further represented through a heatmap constructed using the “ComplexHeatmap” package (v 2.21.1) [21]. Subsequently, the intersection between the DEGs and LCD-associated genes was identified, and this overlapping set was visually presented through a Venn diagram generated by the “ggvenn” package (v 0.1.10) [22].

### 2.3. Machine Learning Screening for Candidate Biomarkers

To improve the robustness of biomarker screening and avoid the bias defect of a single algorithm, this study integrated three complementary machine learning algorithms for analysis. Among them, LASSO regression was used for the regularized feature selection of high-dimensional data, SVM-RFE was adept at identifying optimal classification feature combinations, and random forest relied on ensemble learning to complete feature importance ranking. This study took the intersection of the feature genes screened by the three algorithms as candidate biomarkers, thereby ensuring the cross-algorithm consistency and reliability of the screening results. LASSO regression analysis was performed using the ‘glmnet’ package (version 4.1-8) with alpha = 1 for L1 regularization to screen candidate biomarkers [23]. The optimal lambda value yielding minimal model error was determined via 5-fold cross-validation, based on which LASSO-feature genes were identified. The SVM-RFE algorithm was applied via the ‘e1071’ package (version 1.7-14) [24], likewise employing 5-fold cross-validation. The feature subset demonstrating maximal predictive accuracy was subsequently selected as the optimal combination, with corresponding genes designated as SVM-RFE feature genes. The ‘randomForest’ package (version 4.7-1.1) [25] was employed to construct an RF model based on the identified SVM-RFE feature genes. Numbers of trees from 1 to 100 were tested, and the tree number with the smallest out-of-bag (OOB) error was selected as the optimal parameter; gene importance was calculated using the Gini coefficient, and all genes with importance > 0 were retained as RF-derived feature genes. Finally, potential biological markers were selected from the identified feature genes derived from three machine learning approaches and subsequently visualized via the ‘ggvenn’ package (version 0.1.10).

### 2.4. Expression Level Validation Screening of Biomarkers

The consistency of candidate biomarker expression between the training and validation sets was evaluated via the Wilcoxon rank-sum test, which compared expression levels between the AR and control groups (*p* < 0.05). After identifying the RF-derived feature genes, candidate genes that showed significant differential expression in both datasets and also displayed consistent directional changes were selected for subsequent analysis. Subsequently, the training set underwent ROC curve analysis utilizing the ‘pROC’ package (version 1.18.5) [26], resulting in the calculation of the area under the curve (AUC). Biomarkers exhibiting AUC scores > 0.7 across both datasets were subsequently designated as final markers for further analysis.

### 2.5. Construction of Nomogram

Utilizing the “rms” package (version 6.8-1) [27], a nomogram was built to assess the predictive utility of identified biomarkers for AR. Model calibration was evaluated via calibration curves plotted with the “regplot” package (v 1.1) [28] and confirmed using the Hosmer–Lemeshow test (*p* > 0.05). Decision curve analysis (DCA) was subsequently performed via the “ggDCA” package (v 1.2) [29] to assess the clinical utility of this nomogram, calculating the net benefit across various risk thresholds. Furthermore, ROC analysis was employed to determine the model’s discriminative performance, with AUC values computed via the “pROC” package (v 1.18.5) (AUC > 0.7).

### 2.6. GSEA and GeneMANIA Analysis

GSEA in this study was implemented via the “clusterProfiler” package (version 4.10.1) [30]. The human Gene Ontology Biological Process (GO:BP) gene set categorized as C5 from the MSigDB database was used as the reference. A fixed random seed of 100 was set to ensure the reproducibility of the analytical results. Raw *p* < 0.05, Benjamini–Hochberg (BH)-adjusted FDR < 0.05, and |NES| > 1 were uniformly defined as the screening thresholds for significantly enriched pathways. GSEA was separately performed on biomarkers in the training set. To explore the consistency of biological functions, a Venn diagram was constructed with the “ggvenn” tool (version 0.1.10) to determine overlapping enriched pathways among different biomarkers, with the shared pathways arranged in a sequence based on the magnitude of |NES| in descending order. Additionally, the GeneMANIA database was employed to forecast genes that have functional associations and the biological processes related to these biomarkers.

Additionally, this study performed gene set variation analysis (GSVA) enrichment analysis and calculated enrichment scores for the AR group and control group using the R package GSVA (version 1.46.0) based on the MSigDB database with the c2.cp.v2025.1.Hs.symbols.gmt gene set. Subsequently, the GSVA scores between the two groups were compared in the training set using the Limma package (version 3.58.1), with |t| > 2 as the screening threshold (*p* < 0.05). Spearman correlation analysis combined with BH-adjustment for multiple testing was conducted to investigate the associations of biomarkers with LCD-related pathway enrichment scores, as well as their correlations with LCD-related genes (|cor| > 0.3, adjusted *p* < 0.05).

### 2.7. Chromosome Localization and Subcellular Localization

To define the genetic and cellular characteristics of biomarkers, the “RCircos” package (v 1.2.2) [31] was used for chromosome mapping in conjunction with the GeneCards database. https://www.genecards.org/ (accessed on 12 October 2025) Subcellular Localization Prediction.

### 2.8. Correlation Analysis of Biomarkers and Immune Infiltration Analysis

The interrelationships among the biomarkers were investigated through Spearman correlation analysis utilizing the “ggplot2” package (v 3.5.1) (*p* < 0.05). The “xCell” package (v 1.1.0) [32] was utilized to estimate the enrichment scores of 64 immune and stromal cell types for characterizing the AR immune microenvironment. Differentially enriched immune cell types were defined as cell types with significantly distinct estimated enrichment levels between the AR and control groups; firstly, the BH method was employed to conduct multiple test correction, followed by the Wilcoxon rank-sum test, with a corrected *p* < 0.05 considered statistically significant. Interactions among differential immune cells and between biomarkers and these cells were assessed through Spearman correlation analysis, employing the “psych” package (v 2.4.3) (|correlation coefficient (cor)| > 0.3, *p* < 0.05).

### 2.9. Construction of Molecular Regulatory Network

Transcription factors (TFs) interacting with the identified biomarkers were first retrieved from the TRRUST database and analyzed through the NetworkAnalyst platform to clarify underlying molecular mechanisms and regulatory networks. MicroRNA (miRNA)–biomarker interactions across the DIANA-microT and PITA databases were subsequently predicted using the “multiMiR” package (v 1.24.0) [33], with overlapping miRNAs from these two sources retained for further analysis. An integrated TF–mRNA–miRNA regulatory network was ultimately constructed and visualized through Cytoscape (v 3.9.1) [34].

### 2.10. Potential Drug Prediction for AR

To identify potential therapeutic candidates for AR, compounds associated with biomarkers were searched in the Drug Signatures Database (DSigDB, https://dsigdb.tanlab.org/ (accessed on 13 October 2025). Following this screening process, a potential drug–biomarker interaction network was then built and visualized with the aid of Cytoscape (v 3.9.1).

### 2.11. RT-qPCR

In the Otolaryngology—Head and Neck Surgery Department of the Second Hospital of Shanxi Medical University, clinical specimens were obtained from 5 patients (*n* = 5) with allergic rhinitis and 5 healthy individuals (*n* = 5), and each participant had given their written informed consent. The Institutional Review Board of the Second Hospital of Shanxi Medical University granted ethical approval for this study protocol (Approval No. [2025]YX No. 399).

Total RNA was extracted from five pairs of frozen human tissue samples using Trizol (Servicebio), with three biological replicates each. Subsequently, the extracted RNA was reverse-transcribed into cDNA using the HP All-in-one qRT Master MixII. Subsequently, RT-qPCR was conducted using the 2× SYBR Green qPCR Hub Mix (Servicebio). The primer sequences for prognostic genes are presented in Table 1, with GAPDH acting as an internal control. For each biological sample, all reactions were conducted in triplicate.

### 2.12. Statistical Analysis

The statistical computations were performed using the R software version 4.3.1. Differences between the AR and control groups were assessed using the Wilcoxon test (*p* < 0.05).

### 2.13. Data and Code Availability

The raw expression data used in this study were obtained from the GEO public database. The bioinformatics analysis codes are deposited in the GitHub repository, and the access link is available from the corresponding author. All the processed intermediate datasets are also available upon reasonable request.

## 3. Results

### 3.1. Identification of Candidate Biomarkers

**Figure 1:**
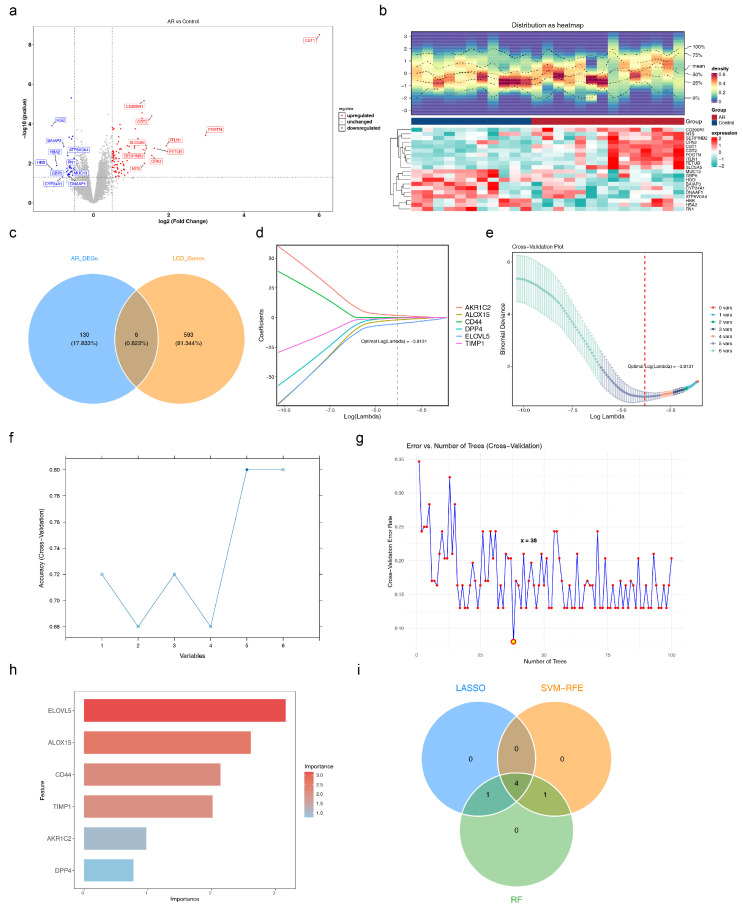
The initial step in this study involved identifying potential markers associated with allergic rhinitis (AR). (**a**) A volcano plot is used to display the expression levels of differentially expressed genes (DEGs) when comparing AR and control samples. In this visualization, red dots correspond to genes that are upregulated, blue dots indicate downregulated genes, and gray dots represent genes that do not show significant changes. In this plot, the red markers correspond to genes that are increased in expression, the blue markers indicate genes with decreased expression, and the gray markers represent genes that do not show significant changes. (**b**) A heatmap is presented to display the expression profiles of the top 10 most upregulated and downregulated differentially expressed genes. (**c**) A Venn diagram is used to display the intersection between DEGs and LCD-associated genes, leading to the identification of 6 potential genes. (**d**,**e**) Through LASSO regression analysis, the coefficient profiles and cross-validation error curve were generated to further analyze the selected features. (**f**) The operational effectiveness and chosen feature genes of the SVM-RFE algorithm are presented here. (**g**,**h**) Random forest model: out-of-bag error and variable importance. Red dots denote cross-validation error rates at different tree counts; the yellow-filled dot highlights the optimal number of trees corresponding to the minimal cross-validation error. (**i**) A Venn diagram illustrating the overlapping feature genes identified through three distinct machine learning approaches.

In the training cohort, 136 DEGs were identified by comparing AR and control samples, including 90 upregulated and 46 downregulated genes in the AR group (Figure 1a,b). Among these 136 DEGs, the intersection with 599 LCD-associated genes yielded 6 potential candidate genes (Figure 1c). LASSO regression analysis identified 5 feature genes (DPP4, AKR1C2, ALOX15, TIMP1, and ELOVL5) at Optimal Log(Lambda) = −3.8131 (Figure 1d,e). Subsequently, the SVM-RFE algorithm was applied to further filter these genes, achieving the highest accuracy of 0.80 when the number of variables was set to 5. This process selected ELOVL5, ALOX15, DPP4, TIMP1, and CD44 as the feature genes identified by SVM-RFE (Figure 1f). In the RF model, the minimum out-of-bag error was observed at ntree = 38, and 6 RF-feature genes were prioritized: ELOVL5, ALOX15, CD44, TIMP1, AKR1C2, and DPP4 (Figure 1g,h). Furthermore, the overlapping feature genes identified through the three machine learning methods also pointed to four potential biomarkers: DPP4, ALOX15, TIMP1, and ELOVL5 (Figure 1i). Furthermore, due to the limited sample size, the machine learning analysis was at risk of overfitting, and the relevant results needed to be interpreted with caution.

### 3.2. Identification of Biomarkers and Their Ability to Distinguish AR and Control Samples

**Figure 2:**
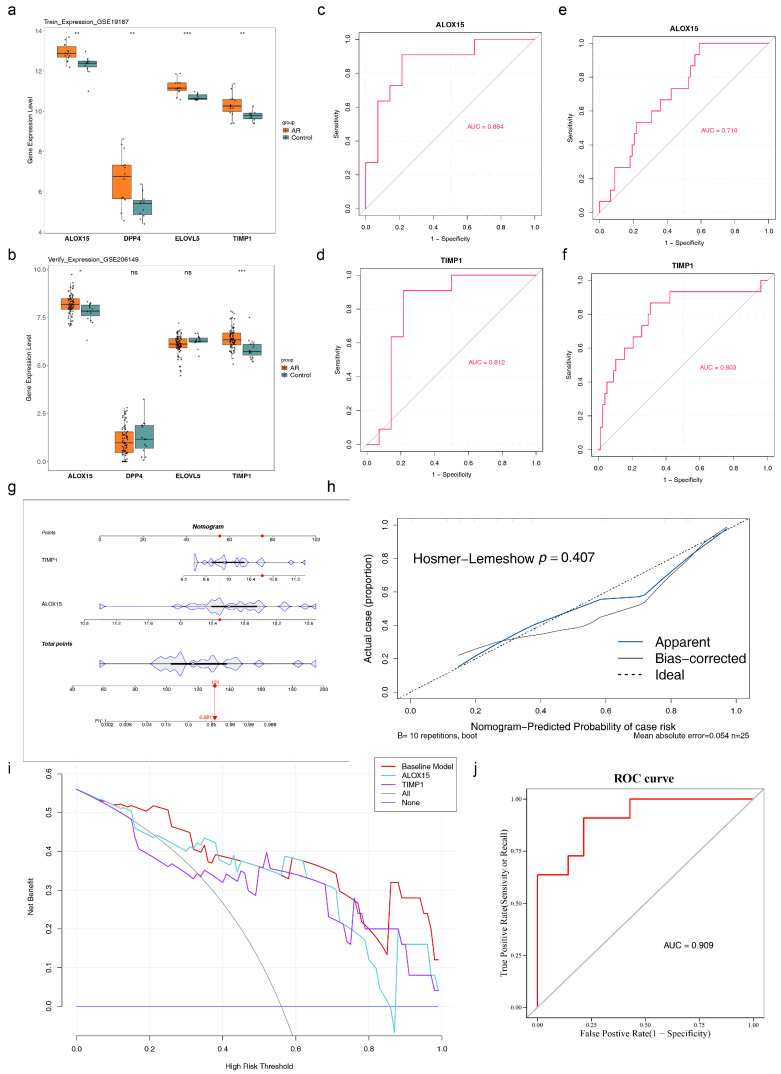
This is followed by the validation of these identified biomarkers and the development of a nomogram to integrate their clinical significance. (**a**,**b**) Validation of potential diagnostic markers in both the training and validation datasets. (**c**,**d**) ROC curves for ALOX15 and TIMP1 were generated using the training cohort to assess their diagnostic performance. (**e**,**f**) ROC curves of ALOX15 and TIMP1 in the validation set. (**g**) A nomogram that predicts the risk of AR by leveraging the expression levels of biological markers. (**h**) The calibration curve of the nomogram model is a graphical representation that assesses the agreement between predicted and observed outcomes. (**i**) Decision curve analysis (DCA) is used to assess the clinical practical value of the nomogram. (**j**) The nomogram model is also assessed via its receiver operating characteristic (ROC) curve. * *p* < 0.05, ** *p* < 0.01, *** *p* < 0.001.

In both the training and validation sets, ALOX15 and TIMP1 were consistently upregulated (*p* < 0.05) (Figure 2a,b). In the training set, the AUC values for ALOX15 and TIMP1 reached 0.864 and 0.812, respectively, both exceeding the 0.7 threshold (Figure 2c,d). In a similar vein, within the validation dataset, the respective AUC figures for these two markers—0.710 for ALOX15 and 0.803 for TIMP1—also stayed above the 0.7 threshold, further confirming their potential as diagnostic indicators (Figure 2e,f). Developed for evaluating AR development likelihood, the nomogram demonstrated a positive association between cumulative scores and disease onset probability. For instance, when the overall score reached 131, the likelihood of developing AR was estimated to be 0.8 (Figure 2g). As shown in Figure 2h, the calibration curve displayed a slope approximating 1. Furthermore, the HL test yielded a *p* value of 0.407, suggesting good model fit and confirming satisfactory predictive accuracy. The clinical applicability of the model is highlighted by DCA results showing a positive net benefit across a broad spectrum of threshold probabilities (Figure 2i). The nomogram showed moderate discriminatory ability between AR and control samples, with an AUC of 0.909 (AUC > 0.7) (Figure 2j).

### 3.3. Enrichment Pathways and Localization Analysis of Biomarkers

**Figure 3:**
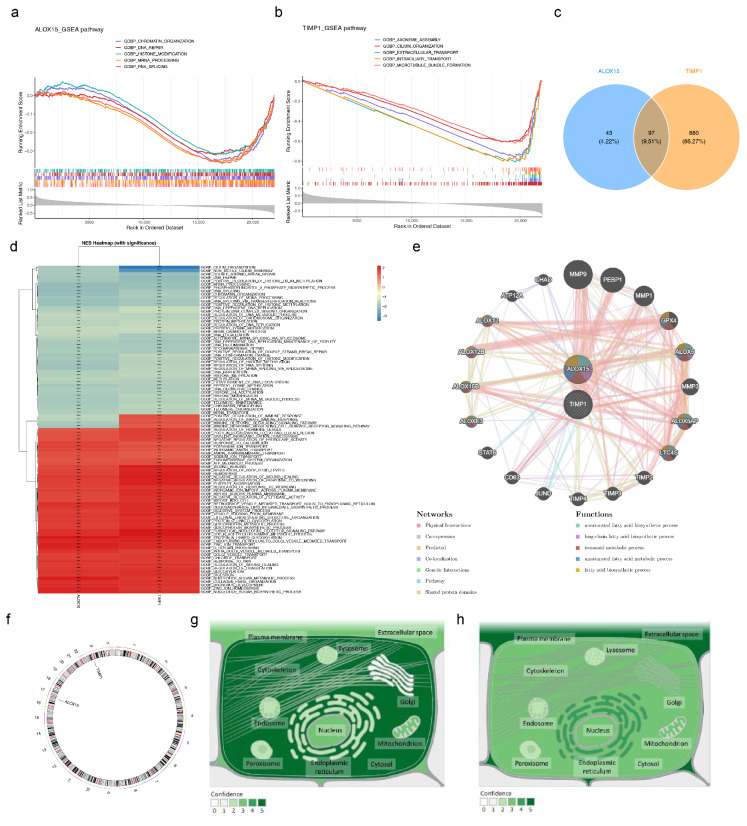
Analyzing the biological functions and subcellular localizations of potential diagnostic or prognostic markers. (**a**) A gene set enrichment analysis (GSEA) also generated an enrichment plot for ALOX15. (**b**) A comparable GSEA enrichment visualization is presented for TIMP1. (**c**) A Venn diagram illustrating co-enriched pathways among ALOX15 and TIMP1. (**d**) A heatmap illustrating shared enriched pathways, with the regulation direction clearly indicated. (**e**) A GeneMANIA network is presented, illustrating functional connections and biological mechanisms. (**f**) The chromosomal positions of ALOX15 and TIMP1 have been determined. (**g**,**h**) Predictions regarding the subcellular positioning of ALOX15 and TIMP1. ** *p* < 0.01, *** *p* < 0.001.

GSEA uncovered notable differences in functional patterns among the biomarkers that were identified. ALOX15 was significantly associated with 140 pathways, while TIMP1 was enriched in 977 pathways (|NES| > 1, *p* < 0.05, and FDR < 0.05). When considering the top pathways associated with ALOX15, which were determined based on their *p*-values, they included chromatin organization, DNA repair, histone modification, mRNA processing, and RNA splicing (Figure 3a, Supplementary Table S2). For TIMP1, the top 5 pathways included axoneme assembly, cilium organization, extracellular transport, intraciliary transport, and microtubule bundle formation (Figure 3b, Supplementary Table S3). A total of 97 pathways were co-enriched by ALOX15 and TIMP1 (Figure 3c). Further analysis of these co-enriched pathways revealed that most of them showed uniform regulatory trends between the two biological markers, while 4 immune-related pathways, including the positive regulation of immune response, regulation of innate immune response, immune response regulating signaling pathway, and immune response regulating cell surface receptor signaling pathway, were uniquely upregulated in TIMP1 (Figure 3d). GeneMANIA network analysis identified 20 closely related genes including MMP9, PEBP1, and MMP1, which primarily interacted through physical interactions and co-expression. These genes were significantly involved in lipid metabolic processes, including unsaturated fatty acid biosynthetic process, long-chain fatty acid biosynthetic process, icosanoid metabolic process, unsaturated fatty acid metabolic process, and fatty acid biosynthetic process (Figure 3e). Genomic localization analysis mapped ALOX15 to chromosome 17 and TIMP1 to the X chromosome (Figure 3f). Complementary subcellular localization forecasts revealed that ALOX15 predominantly resides in the cytoplasmic region and plasma membrane, whereas TIMP1 is mainly positioned in the extracellular matrix (Figure 3g,h). Gathered together, these discoveries offer a comprehensive analysis of the functional connections, regulatory associates, and spatial arrangements of these biological markers, thereby offering essential understandings of their cooperative functions in the development of AR and potential approaches for therapeutic intervention.

In addition, GSVA was conducted in this study to identify four pathways closely related to LCD, including TP53 regulates_transcription of caspase_activators and caspases, RIP_mediated_NFκB_activation_via_ZBP1, Biocarta_CTL_pathway, and medicus_reference_NLRP1_inflammasome_signaling_pathway (Supplementary Figure S1a). Correlation analyses were further carried out between the expression levels of ALOX15 and TIMP1 and the enrichment scores of the above four pathways. The results showed that the score of medicus_reference_NLRP1_inflammasome_signaling_pathway was significantly negatively correlated with ALOX15 (cor = −0.72, *p* = 5.23 × 10^−5^). TP53 regulates transcription of caspase activators, and caspases also displayed a significant negative correlation with ALOX15 (cor = −0.46, *p* = 0.021). By contrast, RIP mediated NFκB activation via ZBP1 was markedly positively correlated with TIMP1 (cor = 0.52, *p* = 0.0071) (Supplementary Figure S1b, Supplementary Table S4). Furthermore, ALOX15 and TIMP1 were separately subjected to correlation analysis with 599 LCD-related genes. The two genes were found to be significantly correlated with 48 and 109 LCD-related genes respectively. Figure 1 illustrates correlation pairs with |cor| > 0.6. ALOX15 had the strongest positive correlation with ELOVL5 (cor = 0.66, *p* = 3.45 × 10^−4^) and the strongest negative correlation with GLUL (cor = −0.63, *p* = 8.28 × 10^−4^). TIMP1 showed the highest positive correlation with CD44 (cor = 0.83, *p* = 3.47 × 10^−7^) and the most obvious negative correlation with GCLC (cor = −0.69, *p* = 1.22 × 10^−4^) (Supplementary Figure S1c, Supplementary Table S5). These findings further verified that ALOX15 and TIMP1 participated in regulating LCD progression and played pivotal roles in the occurrence and development of AR.

### 3.4. Differential Immune Cells in AR

**Figure 4:**
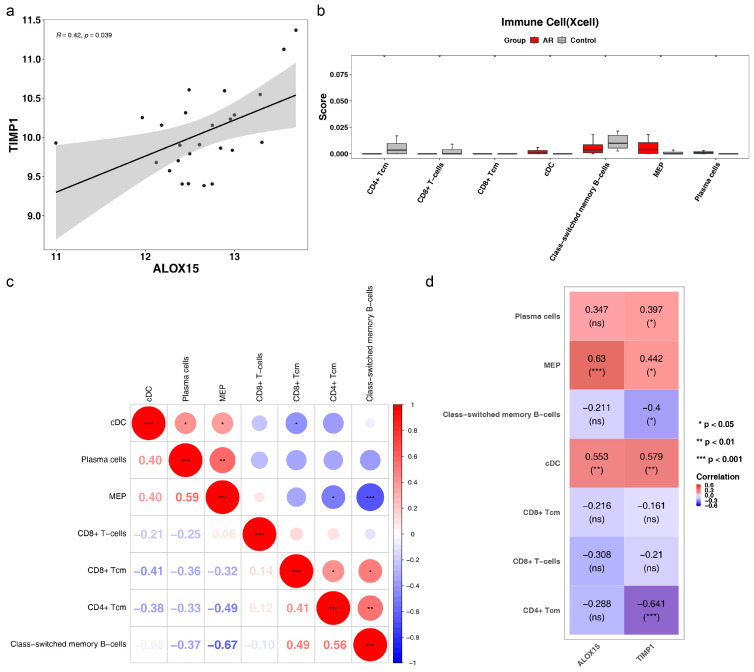
Additionally, exploring the relationship between biological markers and the distribution pattern of immune cells represents another critical aspect in the context of allergic rhinitis (AR). (**a**) The relationship between ALOX15 and TIMP1 was explored through correlation analysis. (**b**) Visualizations of immune cell populations that showed significant differences in abundance when comparing AR patients to healthy controls, presented as box-and-whisker diagrams. (**c**) A correlation matrix is presented for the differential immune cells, where the color coding in the matrix indicates the nature of the relationships: red for positive associations and blue for negative ones. In such visualizations, the color scheme is designed such that a warm tone represents a positive association, while a cool tone indicates a negative relationship. (**d**) A correlation heatmap is presented here, showing relationships between biomarkers and distinct immune cells that exhibit differential abundance.

Correlation analysis revealed a significant positive association between ALOX15 and TIMP1 (cor = 0.42, *p* < 0.05) (Figure 4a). Comparative analysis identified 7 immune cell types with significantly different enrichment scores between the AR and control groups, including CD4+ central memory T cells, CD8+ T cells, CD8+ central memory T cells, conventional dendritic cells (cDCs), class-switched memory B cells, megakaryocyte–erythroid progenitors (MEPs), and plasma cells (*p* < 0.05) (Figure 4b). The MEP enrichment scores showed significant differences between the AR and control groups. Notably, as a semi-quantitative prediction tool, xCell detected significantly higher MEP enrichment scores in the nasal mucosal tissues of AR patients relative to controls, which only suggested potential correlative clues rather than definitive evidence of hematopoietic cell infiltration or lineage reprogramming. These semi-quantitative results required further validation via flow cytometry or single-cell sequencing. Among these differentially enriched immune cell types, the strongest positive correlation was observed between MEP and plasma cells (cor = 0.59, *p* < 0.05), followed by class-switched memory B cells and CD4+ central memory T cells (cor = 0.56, *p* < 0.05). Conversely, the most pronounced negative correlation was detected between class-switched memory B cells and MEPs (cor = −0.67, *p* < 0.05) (Figure 4c). Furthermore, immune cell enrichment score–biomarker correlation analysis indicated that both MEPs and conventional dendritic cells showed strong positive correlations with ALOX15 (cor = 0.63 and 0.53, respectively; *p* < 0.05) and TIMP1 (cor = 0.44 and 0.58, respectively; *p* < 0.05). Plasma cells also exhibited a positive correlation with TIMP1 (cor = 0.40, *p* < 0.05). In contrast, CD4+ central memory T cells and class-switched memory B cells were negatively correlated with TIMP1 (cor = −0.64 and −0.40, respectively; *p* < 0.05) (Figure 4d). These findings indicate potential differences in immune cell enrichment patterns in AR, highlighting correlative associations with disease development.

### 3.5. Molecular Regulatory Mechanisms of Biomarkers and Predictive Drugs for AR

**Figure 5:**
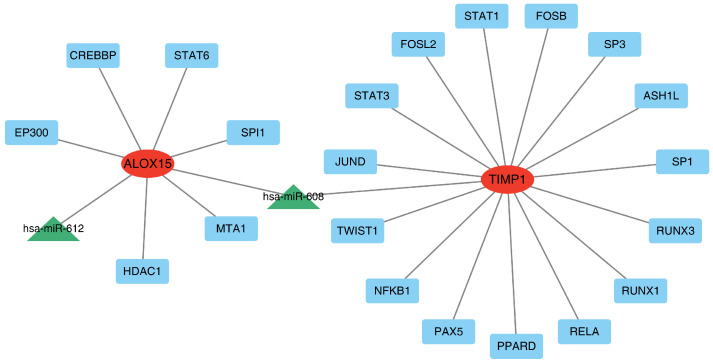
A regulatory network involving transcription factors, microRNAs, and messenger RNAs. The color-coded nodes in the network are defined as follows: red represents biomarkers, blue indicates transcription factors, and green denotes microRNAs.

The regulatory network, which integrated TF–mRNA–miRNA interactions, featured 25 critical nodes connected by 24 interactions. These nodes included the two key indicators, two microRNAs, and 21 regulatory factors. Furthermore, the analysis revealed that ALOX15 was connected to 6 transcription factors, and TIMP1 was associated with 15 transcription factors. Additionally, the microRNA hsa-miR−612 was found to target ALOX15, with this particular miRNA also interacting with both biological markers (Figure 5). Furthermore, in the constructed potential drug–biomarker interaction network, 20 nodes and 22 interactions were detected, where ALOX15 was linked to 14 potential drugs and TIMP1 was associated with 8. Of particular note, four chemical substances (including quercetin, aflodac, NS-398, and fexofenadine hydrochloride) showed connections with both biological markers, as presented in Supplementary Figure S2. This finding provides a bioinformatics-level reference for subsequent targeted drug development targeting these two biomarkers. It should be emphasized that these potential drug–biomarker interactions were merely in silico predictions without subsequent biological validation (e.g., in vitro or in vivo experiments). Therefore, the candidate drugs identified herein should not be regarded as clinically applicable, and their potential efficacy and safety for AR treatment require further experimental verification.

### 3.6. RT-qPCR Experiments of Biomarkers

**Figure 6:**
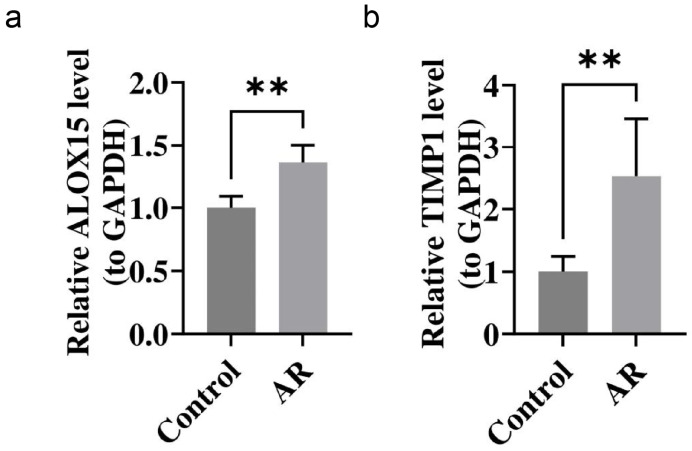
To assess the mRNA expression levels of genes associated with patient prognosis, the reverse transcription–quantitative polymerase chain reaction (RT-qPCR) was employed. (**a**) ALOX15; (**b**) TIMP1. ** *p* < 0.01.

To confirm how these potential indicators behave at the RNA level, researchers carried out RT-qPCR. In line with the bioinformatic predictions, the significant upregulation of ALOX15 and TIMP1 mRNA expression levels in the AR group relative to the control group (*p* < 0.01) (Figure 6a,b) served to confirm the reliability of our bioinformatic analysis.

## 4. Discussion

AR is a common chronic inflammatory condition affecting the nasal lining, predominantly fueled by type 2 cytokines. However, despite these advancements, the identification of consistent indicators for precise individual treatment plans still faces challenges [35]. Research suggests that cells undergoing lytic cell death may release large quantities of intracellular contents and multiple inflammatory factors. Accumulating evidence has demonstrated that intracellular contents released during lytic cell death (LCD) are associated with Th2-type inflammatory responses, epithelial barrier damage, and eosinophil infiltration. Therefore, this process might serve as a potential driving factor in the progression of allergic rhinitis (AR) [36,37]. In the current investigation, two potential markers associated with AR have been discovered. Subsequently, gene enrichment analysis was performed on these biomarkers to explore the signaling pathways and biological functions that they are involved in. Additionally, immune infiltration analysis was carried out to examine the relationship between these biomarkers and the immune microenvironment, whereas subcellular localization analysis was conducted to define their precise intracellular distribution and confirm the validity of their biological functions.

Our study verified that ALOX15 (arachidonate 15-lipoxygenase) was significantly upregulated in AR. ALOX15 is a key enzyme involved in multiple biological processes. It not only influences the start and end of inflammatory reactions but also takes part in cell differentiation, programmed cell death, and the maintenance of oxidative stress balance, thereby playing a crucial part in the development of various pathological conditions. This enzyme’s involvement in disease development is exemplified by its role in respiratory and allergic conditions. For example, in asthma, ALOX15 may influence airway inflammation by producing either pro-inflammatory or anti-inflammatory substances [38]. Similarly, in allergic rhinitis, elevated levels of ALOX15 could promote the release of Th2-type cytokines by boosting the synthesis of pro-inflammatory lipid messengers like 15-hydroxyeicosatetraenoic acid (15-HETE), which in turn worsens nasal inflammation and related symptoms. Additionally, this enzyme is also capable of promoting the process of inflammation resolution by generating anti-inflammatory substances such as lipoxins. Existing literature suggests that ALOX15 is associated with both pro-inflammatory and anti-inflammatory processes in allergic rhinitis [39,40,41]. Furthermore, allergic rhinitis and asthma frequently coexist, representing the clinical concept of the ‘unified airway disease’. In asthmatic individuals, the expression level of ALOX15 in the respiratory tissues is notably elevated, and this enzyme plays a role in regulating eosinophil activity and airway hyperreactivity through comparable pathways, which further substantiates the clinical relevance of ALOX15 in airway allergic diseases [42,43]. Combined with the findings of the present study and relevant literature reports, although ALOX15 can produce both pro-inflammatory and anti-inflammatory mediators, its pro-inflammatory phenotype may tend to predominate in the pathological microenvironment of AR. Notably, there exists technical heterogeneity between the training and validation sets in this study. The training set consists of whole-tissue nasal airway epithelial samples detected by microarray, whereas the validation set comprises nasal brushing samples analyzed via RNA-seq. Despite this heterogeneity objectively enhancing the stringency of validation, the screened biomarkers exhibited consistent expression trends across these two distinct detection platforms and sample sources, further confirming the robustness of their signals. This study found that ALOX15 is significantly upregulated in allergic rhinitis, is closely related to the pathological progression of the disease, and has potential value as a biomarker.

This research work has verified that TIMP1 was also significantly upregulated in AR. TIMP1, also known as tissue inhibitor of metalloproteinases 1, plays a role beyond keeping the extracellular matrix in balance. It is involved in many different normal and abnormal processes, like inflammation, tissue healing, and immune system regulation. Researchers have explored how it functions in various diseases [44]. In allergic rhinitis, previous research has shown that higher levels of TIMP1 might be linked to problems with how matrix metalloproteinases (MMPs) are expressed. This could lead to more tissue swelling and worsen the inflammatory response. TIMP1 expression is correlated with IL-5 levels, eosinophil activation, and mucus secretion, and these indicators are closely associated with the clinical symptoms of allergic rhinitis [45]. In asthma, the increased expression of TIMP1 is closely linked to airway hyperreactivity and airway structural changes [46]. In chronic rhinosinusitis, the balance of TIMP1 and matrix metalloproteinases (MMPs) might influence tissue repair and fibrosis mechanisms, suggesting a potential link between TIMP1/MMP homeostasis and the chronic progression of nasal inflammatory diseases [47]. To summarize, the elevated level of TIMP1 in allergic rhinitis indicates its key involvement in inflammatory reactions and structural changes throughout the disease’s development.

Significantly, the abovementioned ALOX15 and TIMP1, which are actively expressed and functioning, might play a crucial role in connecting the widespread lytic cell death that takes place in AR. On the one hand, intracellular substances released by lytic cell death (e.g., DAMPs) may form a pro-inflammatory microenvironment [15], thereby upregulating the expression of ALOX15 and driving the production of its pro-inflammatory lipid mediators (e.g., 15-HETE). In addition, cell lysis-induced tissue injury might trigger pathological repair mechanisms potentially characterized by an imbalance between TIMP1 and MMP [48]. Conversely, lipid signaling molecules generated by ALOX15 and inflammatory factors regulated by TIMP1 might reciprocally influence the likelihood of lytic cell death in nasal epithelial cells and other cell types. Hence, future studies should focus on investigating the interactions between lytic cell death and the ALOX15/TIMP1 pathways, as understanding such cross-talk could provide insights into the integrated mechanisms governing chronic inflammation and tissue remodeling in allergic rhinitis.

Analysis of gene set enrichment revealed that ALOX15 and TIMP1 may play potential regulatory roles in the pathological development of allergic rhinitis via separate molecular mechanisms. ALOX15 was markedly involved in pathways linked to epigenetic mechanisms, including chromatin organization and histone modification [49]. These biological processes have been demonstrated to control the expression of Th2 cytokines, with histone acetylation even capable of directly boosting the promoter activity of ALOX15 in asthma [38]. This implies that ALOX15 might influence the transcription of genes associated with AR inflammation by adjusting chromatin accessibility, thus sustaining the chronic inflammatory condition of the nasal lining. In contrast, TIMP1 was significantly enriched in pathways related to cellular structure and transport, such as axonemal assembly and ciliary organization. Given that ciliary dysfunction is a common pathological feature of AR and that TIMP1 is also associated with ciliary structural damage in COPD [50], its enrichment in these pathways suggests that TIMP1 may be involved in the maintenance of ciliary stability or intracellular transport through non-classical functions, thereby affecting mucosal barrier and clearance functions [51]. Furthermore, the two genes shared 97 enriched pathways. Notably, four immune-related pathways were uniquely upregulated in the TIMP1 group, suggesting that TIMP1 likely plays a more prominent role in the immune regulation of AR. In subcellular localization, their functions are spatially complementary: ALOX15 mainly resides in the cytoplasm and cell membrane, which aligns with its roles in intracellular lipid metabolism and membrane-associated signal transduction [52]. TIMP1 is located in the extracellular space, which matches its classical function as a secreted protease inhibitor and its role in extracellular matrix remodeling and immune communication [53]. The combined effect of “inside-to-out” (ALOX15 regulating inflammatory signals) and “outside-to-in” (TIMP1 regulating the matrix and immune microenvironment) jointly constituted a potential molecular correlate of persistent inflammation and tissue remodeling in AR.

In the investigation of how biological markers interact with immune cells that exhibit distinct expression patterns, researchers found that ALOX15 could primarily play a role in controlling the body’s natural defense system and stimulating the development of blood cell precursors. Meanwhile, TIMP1 appears to have regulatory functions pertaining to both antibody-based immune responses and the processes related to memory T-cell activation. Furthermore, studies have indicated a positive association between ALOX15 and conventional dendritic cells (cDCs), with existing evidence supporting their roles in the maturation and antigen presentation capabilities of these immune cells. In Th2-type inflammatory states, such as asthma and contact dermatitis, lipid-based signaling molecules produced via the ALOX15 metabolic pathway assist in the movement of cDCs to lymphatic tissues and enhance the polarization of Th2 immune responses [54,55]. Conversely, the observed positive correlation between tissue inhibitor of metalloproteinases 1 (TIMP1) and plasma cells indicates a potential regulatory function in B-cell differentiation and antibody synthesis. This phenomenon has been documented in other immune-related disorders, such as systemic sclerosis and rheumatoid arthritis, which are thought to be connected to the modulation of plasma cell longevity and antibody production [56]. Moreover, research revealed that TIMP1 shows an inverse relationship with CD4+ central memory T cells, implying that it might participate in inhibiting memory T-cell responses, possibly resulting from an impairment of local immune tolerance in AR [57]. Furthermore, investigations into the tumor microenvironment demonstrate that elevated TIMP1 levels are associated with T-cell depletion [58]. In conclusion, ALOX15 and TIMP1 may collectively correlate with the shaping of the transcriptional immune landscape of AR, including a prominent Th2 polarization tendency, plasma cell activation signature, and restrained memory T-cell responses, through their associations with specific immune cell enrichment patterns. These findings provide a reference for future AR therapeutic strategies targeting immune-related molecular signatures. Notably, xCell analysis revealed that the MEP score was significantly elevated in the nasal mucosal tissues of the AR group. MEPs primarily reside in the bone marrow microenvironment, and the biological plausibility of typical hematopoietic MEP cell infiltration in the nasal mucosa is limited. Previous studies have confirmed that the transcriptional characteristics of MEPs are closely associated with proliferation and the TGF-β signaling pathway, which plays a critical role in regulating the fate determination and maturation of erythroid progenitor cells [59,60,61]. Meanwhile, the TGF-β signaling pathway also serves as a core mediator of nasal epithelial remodeling and is markedly abnormally activated in the nasal mucosa of AR patients [62,63]. Therefore, we hypothesize that the elevated MEP score observed in this study does not reflect the actual infiltration of bone marrow-derived hematopoietic MEP cells into the nasal mucosa. Instead, it is more likely that local cells undergo acquired transcriptional reprogramming under the stress of chronic inflammation and injury repair in the nasal mucosa, thereby exhibiting MEP-like transcriptional signatures that are ultimately recognized by the xCell algorithm as an increased MEP signal. In addition, the xCell algorithm only outputs semi-quantitative immune cell enrichment scores, which are susceptible to interference from the transcriptional background and detection platforms and cannot represent the true cellular abundance. The differential enrichment trends of immune cells identified in this study merely represent correlative signals at the transcriptomic level of allergic rhinitis. Further validation through flow cytometry, multiplex immunohistochemistry, and single-cell RNA sequencing is required to characterize the authentic features of the nasal immune microenvironment.

Analyses of transcriptional factors demonstrated that the majority of regulatory proteins linked to ALOX15 and TIMP1 exert crucial control over inflammatory processes and immune system reactions. For example, it is possible that the STAT3 and NF-κB pathways remain activated in the nasal mucosal tissues of patients with allergic rhinitis, which in turn increases the expression of TIMP1 and intensifies the inflammatory response [64]. Importantly, hsa-miR-612 can simultaneously target both ALOX15 and TIMP1, indicating that this microRNA could act as an upstream regulator controlling the combined action of these two genes. As a microRNA with potential regulatory roles, this specific molecule has been found to inhibit tumor growth in both hepatic and pulmonary malignancies, and its reduced levels in certain pathological states tend to enhance inflammatory responses and tissue fibrosis [65]. Hsa-miR-612 can simultaneously target both ALOX15 and TIMP1, and its expression is decreased in allergic rhinitis, which is correlated with the upregulation of these two genes [66]. In terms of potential therapeutic interventions, accumulating basic research has demonstrated that quercetin, a flavonoid derived from natural plants, exhibits prominent anti-inflammatory and free-radical-scavenging activities [67,68,69]. In asthma-related studies, evidence has shown that quercetin can inhibit ferroptosis in asthma models, reduce M1-type macrophage polarization in neutrophil-dominant airway inflammation, downregulate TIMP1 expression, and ameliorate the process of airway remodeling [67,68,69]. However, it must be pointed out that these findings are preliminary and based on in silico predictions. Direct in vitro or in vivo evidence confirming the efficacy of quercetin in AR models is currently lacking. In addition, the clinical application of quercetin is significantly limited by its poor oral bioavailability and rapid systemic clearance. Therefore, although this computational analysis proposes quercetin as a candidate compound worthy of further investigation, it should not be interpreted as an immediate clinical recommendation. Future studies using specific AR cell lines or animal models are critical to validating the functional interaction between quercetin and the ALOX15/TIMP1 axis, which will be essential before considering its therapeutic application.

## 5. Conclusions

Using public transcriptomic datasets and lytic cell death-related genes, this study integrated differential expression analysis, machine learning, and experimental validation to identify ALOX15 and TIMP1 as key biological markers for AR. Bioinformatic analyses revealed that both genes are jointly involved in the regulation of the immune microenvironment in AR: ALOX15 is mainly implicated in epigenetic and inflammatory pathways, while TIMP1 is associated with cellular structure and extracellular matrix remodeling. Significantly, these two genes work together to influence the immune system’s response within AR, with ALOX15 primarily playing a role in modifying genetic expression patterns and triggering inflammatory processes, whereas TIMP1 contributes to maintaining cell structure integrity and facilitating changes in the surrounding extracellular matrix. Subsequent research into the upstream regulatory network and possible drug predictions has revealed their underlying molecular mechanisms and therapeutic applications, providing a theoretical basis for exploring AR pathogenesis and potential therapeutic strategies. This study has several limitations. First, this study mainly screened potential LCD-related biomarkers by combining transcriptomic data from public databases with machine learning approaches. It lacks experimental validation such as Western blotting and immunohistochemistry; thus, it can only indicate gene associations at the transcriptomic level and cannot directly confirm the occurrence of LCD in nasal mucosa. Second, restricted by the clinical information of public datasets, this study did not perform stratified analyses according to AR subtype, disease severity, or allergen profile, nor could it exclude the confounding interference of medications (e.g., intranasal glucocorticoids, antihistamines, and allergen-specific immunotherapy) on gene expression. Third, only five samples were included in each group for RT-qPCR validation, resulting in a small sample size. Future studies should rely on multicenter large-sample cohorts to further verify the generalizability of ALOX15 and TIMP1 as AR biomarkers. Fourth, the training set was derived from microarray data, whereas the validation set was based on RNA-seq data, leading to significant technical heterogeneity. Subsequent validation should be performed using multicenter cohorts with a unified detection platform and standardized sampling protocol. Fifth, this study confirmed that ALOX15 is upregulated in AR; however, the functional balance between its pro-inflammatory and anti-inflammatory roles in the inflammatory microenvironment remains unclear. In future work, detecting the 15-HETE/lipoxin ratio in nasal lavage fluid combined with single-cell RNA sequencing to analyze its cell-type-specific expression patterns may help clarify its net biological effect. Sixth, most results are limited to bioinformatic prediction and preliminary experimental verification. Functional experiments such as gene knockout and overexpression in cell and animal models have not yet been conducted. The regulatory networks and drug predictions are merely computational outcomes, and their regulatory relationships and drug efficacy still require experimental validation. Seventh, the cohort in this study strictly excluded patients with atopic comorbidities and nasal complications. Nevertheless, comorbid allergic diseases are common in real-world clinical practice, and different comorbidity statuses may lead to distinct type 2 inflammatory responses and molecular expression profiles. Large-sample stratified cohorts are needed to further explore their effects on AR inflammatory characteristics and biomarkers. In view of the above limitations, we plan to construct a prospective clinical cohort in follow-up work, enrolling treatment-naïve AR patients without prior anti-allergic or immunological interventions. Detailed stratification by AR subtype, disease severity, and allergen type will be performed to eliminate drug-related confounding. Meanwhile, large-sample stratified cohorts will be used to dissect the regulatory effects of atopic comorbidities on the inflammatory landscape and biomarkers of AR. These efforts will gradually improve the study design and promote the clinical translation of our research findings. Notwithstanding these drawbacks, this research certainly offers potential new markers and a robust theoretical foundation for the precise diagnosis, therapy, and targeted medication development of AR.

## Figures and Tables

**Table 1:** Primers for validation of biomarkers.

Primer	Sequence (5′-3′)
ALOX15 F	GCTGTGAAAGACGACCCAGA
ALOX15 R	GCACCCAAGAGTACCAGTCC
TIMP1 F	CATCCGGTTCGTCTACACCC
TIMP1 R	GTGCATTCCTCACAGCCAAC
GAPDH F	ATGGGCAGCCGTTAGGAAAG
GAPDH R	AGGAAAAGCATCACCCGGAG

## Data Availability

The raw data supporting the conclusions of this article will be made available by the authors on request.

## Supplementary Materials

The following supporting information can be downloaded at: https://www.mdpi.com/article/10.3390/biomedicines14061284/s1. Supplementary Figure S1. Correlation of ALOX15 and TIMP1 with LCD-related pathways and genes. (a) GSVA pathways related to LCD. (b) Correlation analysis between biomarkers and pathways. (c) Correlation between biomarkers and LCD-related genes. Supplementary Figure S2. The drug–biomarker interaction network is presented here, where the color-coded components are defined as follows: red-orange nodes represent biomarkers, and green nodes indicate in silico-predicted medications. Supplementary Table S1. A compilation of genes associated with lytic cell death processes. Supplementary Table S2. The GSEA results for ALOX15 have been fully completed. Supplementary Table S3. The GSEA analyses for TIMP1 have been fully completed. Supplementary Table S4. Correlation analysis between biomarkers and pathways. Supplementary Table S5. Correlation between biomarkers and LCD-related genes.

## Abbreviations

The following abbreviations are used in this manuscript:

AR	Allergic rhinitis
LCD	Lytic cell death
HMGB1	High-mobility group box 1
DAMPs	Damage-related molecular patterns
Th2	T helper 2
ILC2s	2 innate lymphoid cells
IL-33	Interleukin-33
ROC	Receiver operating characteristic
GEO	Gene Expression Omnibus
DEGs	Differentially expressed genes
AUC	Area under the curve
DCA	Decision curve analysis
GSEA	Gene set enrichment analysis
TIMP1	Tissue inhibitor of metalloproteinases 1
ALOX15	Arachidonate 15-lipoxygenase
MMPs	Matrix metalloproteinases
cDCs	Conventional dendritic cells
TFs	Transcription factors
miRNA	MicroRNA
DSigDB	Drug Signatures Database
15-HETE	15-hydroxyeicosatetraenoic acid
IL-5	Interleukin-5
MMP	Matrix metalloproteinase
