# Supplementary figures and images for: Exploring Biomarkers and Regulatory Mechanisms Associated with Lytic Cell Death in Allergic Rhinitis Based on Transcriptome Analysis
Source: Biomedicines. 2026 Jun 4;14(6):1284. doi: 10.3390/biomedicines14061284 (PMC13296438; doi:10.3390/biomedicines14061284)

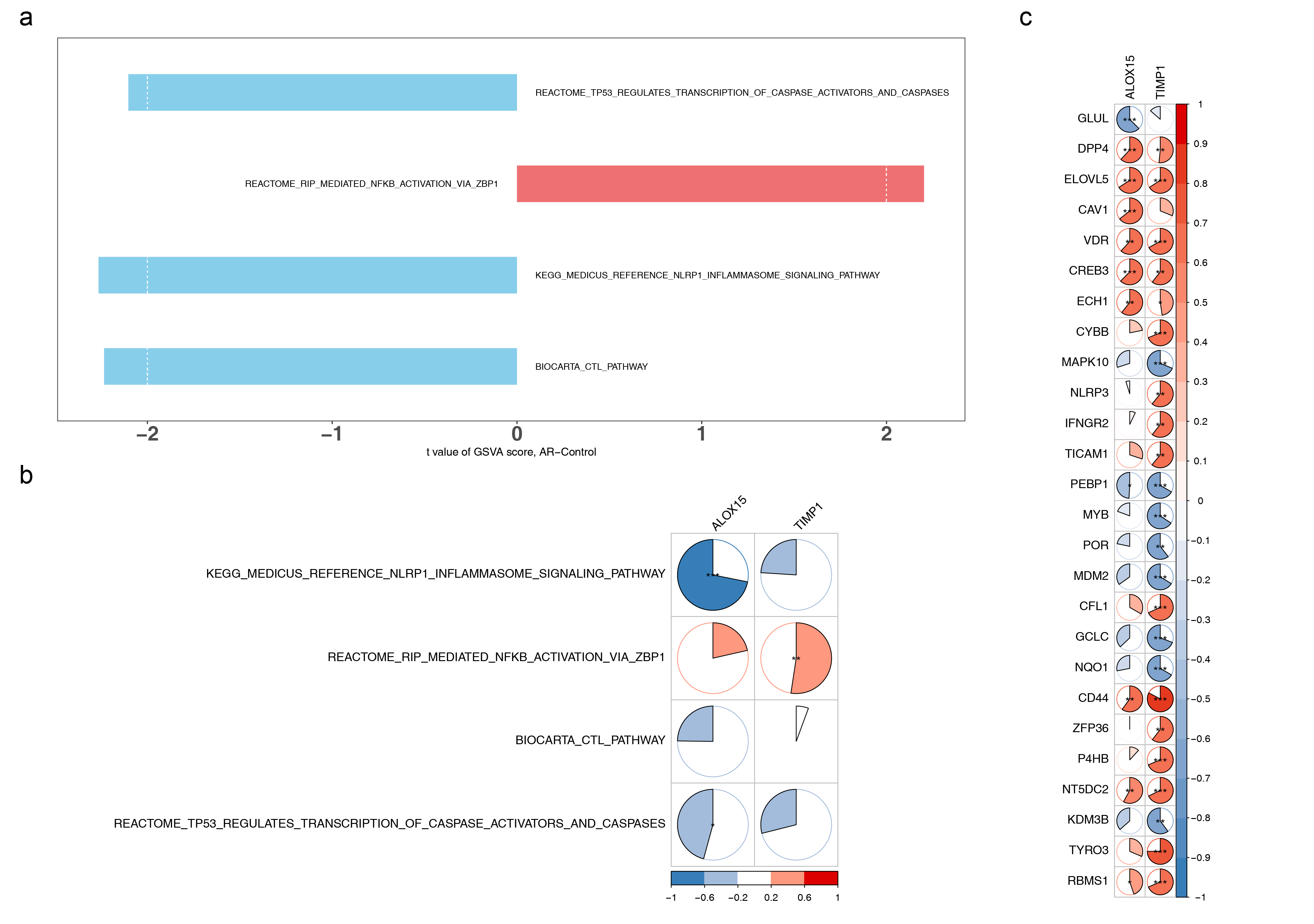

Supplement: Supplementary file 1 [file biomedicines-14-01284-s001.zip › Figure S1.tif]

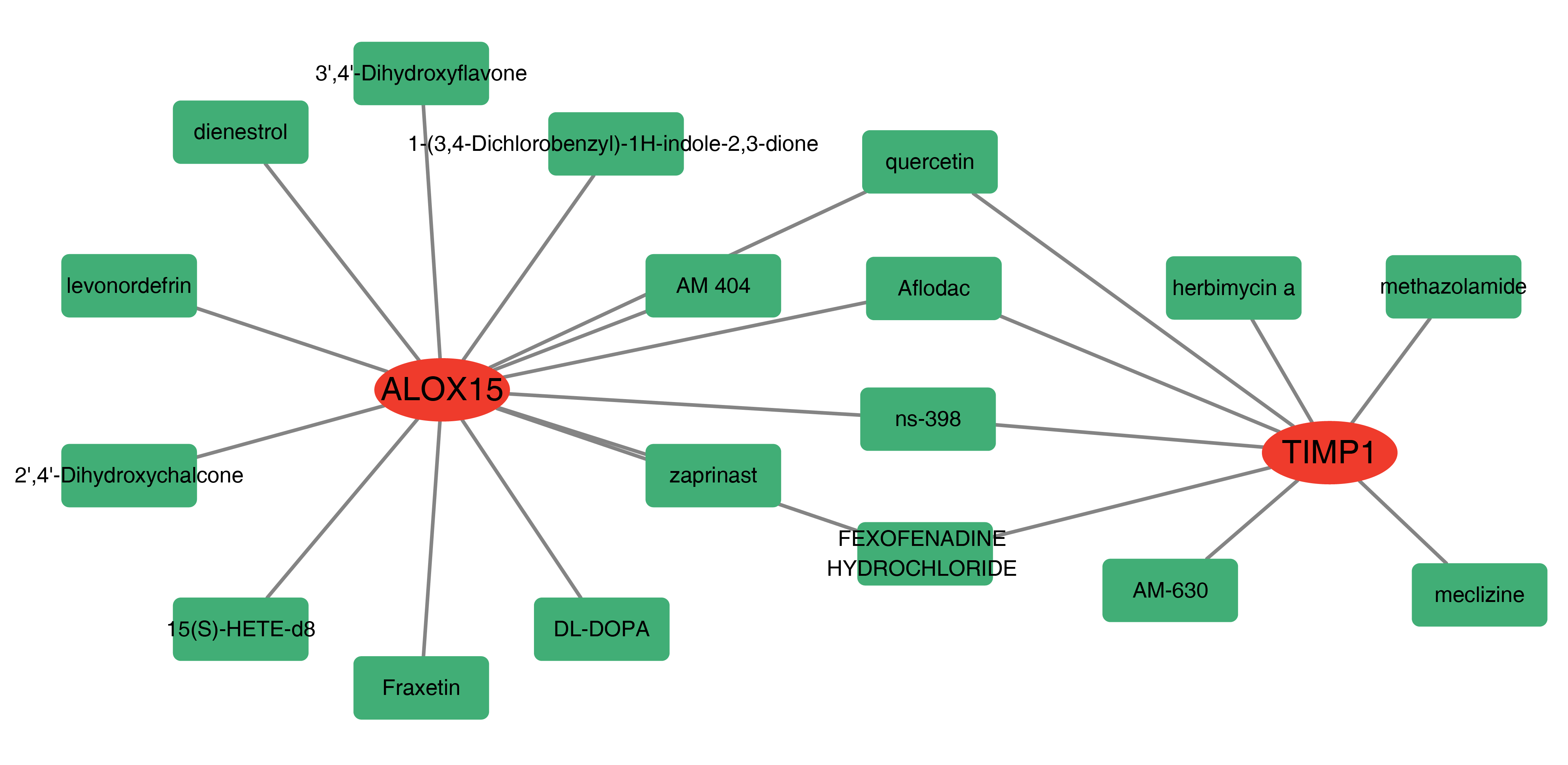

Supplement: Supplementary file 1 [file biomedicines-14-01284-s001.zip › Figure S2.tif]
